# A Longitudinal Study on the Emotional Impact Cause by the COVID-19 Pandemic Quarantine on General Population

**DOI:** 10.3389/fpsyg.2020.565688

**Published:** 2020-09-18

**Authors:** Lorena Canet-Juric, María Laura Andrés, Macarena del Valle, Hernán López-Morales, Fernando Poó, Juan Ignacio Galli, Matías Yerro, Sebastián Urquijo

**Affiliations:** ^1^Institute of Basic and Applied Psychology and Technology (IPSIBAT), National University of Mar del Plata (UNMDP), Mar del Plata, Argentina; ^2^National Scientific and Technical Research Council (CONICET), Buenos Aires, Argentina

**Keywords:** coronavirus, COVID-19, emotion, mental health, anxiety, depression, longitudinal, quarantine

## Abstract

COVID-19 pandemic represents, not only a public physical health emergency, but a mental health serious problem as well. However, little is known about the psychological impact of the quarantine during this pandemic. The aim of this study is to assess the emotional impact of the lockdown measures imposed by the Argentinian government to fight the virus. For this, a survey was distributed on social network. We surveyed the Argentinian general population twice: 2 days after the mandatory quarantine started (time 1), and 2 weeks later (time 2). Anxiety levels were assessed using the State-Trait Anxiety Inventory; depressive symptoms were assessed using the Beck Depression Inventory-II; and affect was assessed using the Positive and Negative Affect Scale. A total of 6057 people answered both surveys. In addition, different socio-demographic factors were considered, such as risk factors for COVID-19, age, gender, educational level, variation in family income due to quarantine, number of children, whether they have older adults in charge or not and the number of hours viewing information about COVID-19. Statistically significant variations were observed between the two time points. The effect size, however, was very small. Depression tends to increase slightly, while levels of anxiety and affect (positive and negative) tend to decrease. Also, some slight differences related to the socio-demographic factors were found. Findings suggests that sustaining the lockdown measures could have a larger effect on mental health in the long term. It is necessary to continue monitoring emotional distress and other related mental health problems on the general population. It is also necessary to create programs aimed at promoting mental health, and to distribute information about it.

## Introduction

On March 3, Argentina confirmed its first case of COVID-19. As of March 20, 2020, given the epidemiological situation and with a total of 128 confirmed cases of COVID-19 in the country, the Argentinian government established “social, preventive and mandatory isolation,” restricting mass circulation (excluding people affected by essential activities and services). Suddenly, people can only travel for essential issues, such as buying food, cleaning supplies or medicines. On March 22, that is, 2 days after the official quarantine began, we initiated a longitudinal psychological study. We started data collection in order to analyze the emotional impact of quarantine on people. For this purpose, online surveys were used to assess basic affective and psychological dimensions (i.e., depressive symptoms, anxiety levels, and positive and negative affect) that could be compromised in this context. Explanatory or moderating factors (e.g., gender, age, risk factors for COVID-19, etc.) were also explored. Two weeks after quarantine began, participants were contacted again to complete a second version of the survey.

In general, quarantine has been described as an unpleasant experience for those who undergo it, because it may involve separation from loved ones, financial problems, uncertainty over the situation and boredom, among other consequences (Cava et al., 2005; Brooks et al., 2020). Furthermore, emotional problems and lost income led the list of the main problems associated with quarantine during the SARS outbreak in Toronto (Blendon et al., 2004). Also, Hawryluck et al. (2004) reported that quarantine may be associated with significant psychological distress, depressive symptoms and post-traumatic stress.

Anxiety and depression are affective responses that serve important adaptive functions. However, the recurrence, persistence and intensity of these responses can hinder psychosocial and physiological functioning. Depression, related to grief or sadness, occurs after real or perceived loss (Beck et al., 1996; MacKinnon and Hoehn-Saric, 2003). Anxiety is an emotional state characterized by subjective feelings of tension and apprehension, as well as autonomic nervous system responses (Spielberger et al., 1999). On the other hand, positive affect is the degree to which a person feels satisfied, enthusiastic, energetic, active and alert. On the contrary, negative affect refers to subjective distress and involves a variety of aversive emotional states, such as anger, contempt, disgust, guilt, fear, or nervousness (Watson et al., 1988).

A recent review (Brooks et al., 2020) on 26 studies, shows negative psychological effects of quarantine, including post-traumatic stress symptoms, confusion and anger. Stressors included prolonged quarantine durations, fears of infection, frustration, boredom, lack of supplies, inadequate information, among others. Some studies have even suggested lasting effects after the quarantines had ended. Research conducted in countries with early spread of the COVID-19 have revealed the wide psychological impact and its consequences for people (Lima et al., 2020). As reported, psychological symptoms may emerge in individuals without previous mental disorders, or worsen in those with pre-existing psychological conditions. It is also possible that anguish emerge (or increased) among infected people or those who care for them (Kelvin and Rubino, 2020). Also, the quarantine can elicit serious distress among people and consequently increase the suicide rates in general population, or in health-care workers (Ammerman et al., 2020; Goyal et al., 2020). Similarly, Barbisch et al. (2015) indicate that quarantine can cause collective hysteria, leading hospital staff to take desperate measures.

According to Brooks et al.’s (2020) recent review, there is only one study, of the 26 considered, about the longitudinal psychological effects of quarantine. The study found that 7% of people showed anxiety symptoms and 17% showed feelings of anger, but 4–6 months after quarantine had ended, these symptoms had decreased to 3 and 6%, respectively. Regarding long-term effects, some studies indicate that 3 years after a SARS outbreak, some health-care workers still reported problematic alcohol use or dependence symptoms (see Brooks et al., 2020).

It has also been pointed out that after a period of quarantine, health-care workers continued to engage in avoidance behavior such as limiting direct contact with patients and not reporting to work (Cava et al., 2005). In summary, most of these studies indicate that, after a prolonged period of quarantine, some people exhibited social avoidance behaviors, mental health problems, and post-traumatic stress disorder, among other problems. However, a recent longitudinal study (Wang et al., 2020) on the COVID-19 pandemic did not report significant changes in levels of anxiety and depression. The COVID-Minds Network (funded by Wellcome Trust) aims to support the development of quality longitudinal COVID-19 studies in different countries around the world, exploring the effects of the pandemic on mental health. On its website, the network synthesizes some of the preliminary findings: (1) The mental health of the population during pandemic lockdown has worsened from previous measures, and could potentially return to pre-pandemic levels as lockdown restrictions are lifted; (2) the pandemic does not affect everyone equally (e.g., younger adults and females have been experiencing worse psychological responses); (3) people’s health behaviors are been affected by the pandemic as well (Covid-Minds Network, 2020).

In addition, some studies report that certain socio-demographic factors moderate the psychological impact of quarantine. A particular study (Taylor et al., 2008) found that gender, age, number of children and educational level, were aspects associated with the psychological effect of the quarantine. However, other studies (e.g., Hawryluck et al., 2004) indicate that demographic factors such as marital status, age, educational level, living with other adults and having children were not associated with psychological effects during quarantine.

In general, over 100 countries worldwide had instituted either a full or partial lockdown by the end of March 2020, affecting billions of people. Some of the more common approaches have been government recommendations on social distancing (localized or general recommendations). Others have opted for restricting all non-essential internal movement (lockdown). In Europe, for example, almost all countries (except five) have had some period of national lockdown. Meanwhile, Asia, Australia, and New Zealand, among other countries, have adopted a national or local lockdown approach. Some others have adopted mixed approaches (that include periods of social distancing and periods of more controlled lockdown). Finally, in the Americas, where the appearance of the first confirmed cases of COVID occurred later, there are various positions, such as Brazil that has opted for localized lockdown or Uruguay that has opted for national recommendations (Dunford et al., 2020). The actions taken by the Argentinian government in response to the COVID-19 (general lockdown), installed the need to investigate how quarantine impacts on people’s emotional state. Researching the behavioral and emotional changes of people in these situations is essential in order to provide tools to the public health system. The findings can help to plan remedial measures, as well as to identify aspects that require further analysis, to recognize possible severe psychological distress and to know how to act in possible future similar situations. Knowing and understanding the experiences of people in quarantine has been highlighted as a central tool to maximize control over the spread of the disease, as well as to minimize the negative effects on affected individuals, families and communities (Hawryluck et al., 2004). Therefore, the aim of this study is to analyze the longitudinal emotional effect of the social, preventive and mandatory isolation established due to the epidemiological COVID-19 situation in Argentina.

## Materials and Methods

### Participants

On March 22nd, that is, 2 days after the lockdown started, the survey was launched. Fourteen days after, a second survey was sent to all the people that had completed the first one. A total of 6057 people participated in both the first evaluation (between March 22nd and 25th) and the second evaluation (between April 3rd and 9th; that is, 12–15 days between them). Out of the 6057, 91.6% of the respondents were affected by isolation measures, and 508 (8.4%) were exempted (health workers, laboratory technicians, security forces personnel, people from the agricultural sector, veterinarians, media workers, pharmacists, food sale and delivery workers, public government staff, researchers, machine operators, among others). Participants were mostly between the ages 18–40 (63.3%), quarantined at home with other people (83.7%), worked regularly (80.2%), perceived the quarantine had little or no economic impact on them (62,1%), and lived in a spacious house (40.3% lived in a house with 4 or more rooms) with available open space (75.7% said they have a garden or a backyard). The main socio-demographic characteristics of the sample are described in Table 1. The inclusion criteria were to be over 18 years old, to live in Argentina, and to no suffer from physical or psychological illnesses.

**TABLE 1:** Socio-demographic characteristics for all sample.

**Variable**		***n***	**%**
		**6057**	**100**
Risk factor for	Yes	1179	19.5%
COVID-19	I don’t know	219	3.6%
	No	4659	76.9%
Perceived degree	10–40%	41	0.6%
of quarantine	50–70%	133	2.3%
compliance	80–100%	5833	97.1%
Age group	18–25	929	15.3%
	26–40	2910	48.0%
	41–60	1803	29.8%
	60 or +	415	6.9%
Gender	Female	4886	80.7%
	Male	1131	18.7%
	Other	20	0.3%
	Prefer not to answer	20	0.3%
Educational level	Postgrad	1696	28%
	University (complete)	2120	35%
	University (incomplete)	1823	30.1%
	Secondary (complete)	342	5.6%
	Secondary (incomplete)	69	1.1%
	Primary (complete)	6	0.1%
	Primary (incomplete)	1	0.0%
Perception of economic impact	No	2666	44%
	Few	1094	18.1%
	Some	983	16.2%
	Much	320	5.3%
	Very much	726	12%
	Other responses	268	4.4%
Work regularly	Yes	4858	80.2%
	No	1199	19.8%
Number of children	0	3202	52.9%
	1	960	15.8%
	2	1206	19.9%
	3	495	8.2%
	4 or more	194	3.2%
Alone or accompanied during quarantine	Alone	987	16.3%
	Accompanied	5070	83.7%
Older adults in charge	No	2260	37.3%
	Yes, living with me	1761	29.1%
	Yes, living somewhere else	1240	20.5%
	Yes, but partially and living somewhere else	796	13.1%
Number of rooms	1 (studio apartment)	153	2.5%
	2	1584	26.2%
	3	1794	29.6%
	4 or more	2443	40.3%
	Other responses	83	1.4%
Presence of outdoor spaces	None	416	6.9%
	Balcony, terrace	1058	17.5%
	Garden, backyard	4583	75.7%
Daily news hours	Few (less than 1 h)	183	3.0%
	Regularly (2 h)	4292	29.00%
	Much (3 or 4 h)	2943	19.9%
	All day (4 h or more)	1997	13.5%
	Other responses	510	3.5%

### Measures

#### Depressive Symptoms

The Spanish adaptation (Sanz et al., 2005; Sanz and Vázquez, 2011) of the Beck Depression Inventory-II (BDI-II) (Beck et al., 1996) was used. The BDI-II is a self-report measure that provides information about the presence and severity of depressive symptoms. BDI-II consists of 21 items indicating symptoms such as sadness, loss of pleasure, feelings of failure and guilt, pessimism, etc. People have to answer questions about how they felt in the past two weeks, to be consistent with the DSM-IV criteria for major depression. Participants rated items on 0–3 scales, with higher scores representing more severity of the symptom. The BDI-II has adequate reliability (α = 0.89, Sanz et al., 2003) and validity (e.g., Sanz and Vázquez, 1998; Beltrán et al., 2012). Item 9 (suicidal ideation) was omitted for this study due to the potential risk it might imply in online surveys.

#### State Anxiety

The Spanish version (Spielberger et al., 1999) of the State-Trait Anxiety Inventory (STAI; Spielberger et al., 1970) was used. The STAI is a self-report measure composed of 40 items which assess anxiety as a transitory state and as a stable trait. In this study we only used the state-anxiety dimension, which is composed of 20 items answered on 4-point Likert scale (from 0 to 3). In Spanish population, internal consistency range from α 0.84 to 0.93 (Riquelme and Casal, 2011).

#### Positive and Negative Affect

The Spanish adaptation (López-Gómez et al., 2015) of the Positive and Negative Affect Schedule (PANAS; Watson et al., 1988) was used. The PANAS includes two subscales, Positive Affect and Negative Affect, each of one contain 10 items such as “tense,” “nervous,” or “satisfied.” The participant is asked to indicate whether he or she is feeling that way at the moment. Items are rated on a 5-point Likert scale (from 1 = *not at all* to 5 = *very much*). In Spanish population, internal consistency range from α 0.83 to 0.92 (López-Gómez et al., 2015).

#### Socio-Demographic Factors

Closed questions were included. We asked about (a) Perceived degree of quarantine compliance, (b) Risk factors for COVID-19, (c) Age, (d) Gender, (e) Variation in economic income due to quarantine, (f) Educational level, (g) Employment, (h) Number of children, (i) Whether he or she is alone or accompanied during quarantine, (j) Number of rooms in the quarantine site, (k) whether or not the respondent has dependent older adults, (l) Presence of outdoor spaces in the quarantine site, and (m) hours a day consuming news. All these socio-demographic aspects were assessed only once in time 1.

### Procedure and Ethical Considerations

The data collection was done through Google Forms. The first freely access survey (Time 1; T1) was disseminated by social networks between March 22 and 25 (close to the beginning of isolation measures in Argentina). Twelve to fifteen days later (between April 03 and 09, depending on the day they had answered the first one), we contacted people again and sent them the second survey (Time 2; T2). For this research, all the procedures were conducted in accordance with the ethical standards of the Helsinki Declaration and the CONICET guidelines for ethical behavior in Social Sciences. People participated voluntary and only after singing (digitally) an informed consent. We provide the contact information of the research group in order to answer any question that may arise regarding the rights of research subjects. The study was approved by the Interdisciplinary Thematic Program in Bioethics of the National University of Mar del Plata.

In addition, we considered the potential risk of conducting online surveys (without researcher’s direct supervision) in the context of quarantine. In this regard, evidence (Jorm et al., 2007; Yeater et al., 2012) indicates that only a very small portion of participants experience distress when answering questions about their mental health, trauma, or adverse experiences. Thus, it has been suggested that answering online surveys would not have negative short-term effects event when investigating sensitive issues such as the presence of self-injurious behavior (Muehlenkamp et al., 2015). In fact, positive reactions are generally more common than negative ones (Jorm et al., 2007), and even those who report some kind of negative reaction during the study, judge their participation as positive (Jorm et al., 2007; Tan et al., 2019). In any case, participants were provided with information about different psychological support devices to which they could turn if necessary. We also emphasized that the participant could stop answering at any time.

### Statistical Analysis

Reverse item were recoded and the dimensions of anxiety, depressive symptoms and affect (positive and negative) were calculated. Descriptive statistical analyses were applied to characterize the sample. Subjects were grouped according to their socio-demographic features. Some of the closed questions categories were grouped to improve understanding of the results. Repeated measures *ANOVA* statistic was used to test for differences between the first and the second surveys. Sociodemographic variables (Table 1) were considered as the inter-subject factor and time-point as the intra-subject factor. Regarding gender and educational level, some groups had to be excluded from the inferential analyses due to the small sample size. Partial eta square was used to analyze effect size. The *Bonferroni* statistic was used for intergroup and intragroup multiple comparisons. Interaction effects were also graphically presented.

## Results

### Changes in Depressive Symptoms Between the First and the Second Survey

Descriptive statistics for depressive symptoms are presented in Table 2. In the first place, we conducted a repeated measures ANOVA considering all the sample. The statistic showed a significant difference (*F* = 98.84; *p* < 0.001) between T1 and T2, however, the effect size of this difference was very small ($\eta_{\text{p}}^{2}$ = 0.016). In the second place, we conducted the ANOVAs considering the socio-demographic variables as the inter-subject factor and time-point as the intra-subject factor. Results are presented in Table 3.

**TABLE 2:** Descriptive statistics for depressive symptoms in Time 1 and Time 2.

**Depression**		**Time 1**		**Time 2**	
		**ME**	***SD***	**ME**	***SD***
All sample (*n* = 6057)		8.74	7.41	9.41	7.88
Do you quarantine?	Yes	8.78	7.42	9.46	7.88
	Exempted/no	8.31	7.32	8.77	7.87
Risk factor for COVID-19	Yes	8.20	7.54	8.76	8.07
	I don’t know	11.13	8.72	11.66	8.96
	No	8.77	7.29	9.46	7.75
Perceived degree of quarantine compliance	10–40%	8.61	5.63	10.22	9.19
	50–70%	10.26	8.48	10.73	8.68
	80–100%	8.71	7.39	9.37	7.85
Age group	18–25	12.43	8.95	13.29	9.32
	26–40	9.04	7.24	9.74	7.76
	41–60	7.11	6.30	7.68	6.71
	60 or +	5.50	5.41	5.86	5.82
Gender	Female	9.14	7.53	9.82	7.97
	Male	6.95	6.42	7.50	7.03
	Other	14.45	11.21	15.75	11.95
	Prefer not to answer	8.40	8.22	8.95	7.80
Educational level	Postgrad	7.30	6.31	8.15	6.90
	University (complete)	8.17	6.75	8.79	7.25
	University (incomplete)	10.36	8.22	10.97	8.69
	Secondary (complete)	10.62	9.43	10.69	9.62
	Secondary (incomplete)	10.14	8.15	11.71	9.32
	Primary (complete)	–	–	–	–
	Primary (incomplete)	–	–	–	–
Perception of economic impact	No	7.70	6.68	8.44	7.27
	Few	9.07	7.18	9.66	7.95
	Some	9.24	7.38	9.98	7.81
	Much	10.70	8.47	11.03	8.55
	Very much	10.77	8.83	11.18	8.99
Work regularly	Yes	8.38	7.06	9.07	7.56
	No	10.24	8.52	10.77	8.93
Number of children	0	9.91	7.96	10.66	8.44
	1	8.44	7.11	8.98	7.38
	2	7.15	6.12	7.82	6.73
	3	6.51	6.00	7.03	6.35
	4 or more	6.61	6.24	6.71	6.75
Alone or accompanied during quarantine	Alone	8.60	7.43	9.07	7.95
	Accompanied	8.77	7.41	9.47	7.86
Older adults in charge	Yes	10.82	8.65	11.18	8.98
	No	8.65	7.40	9.32	7.87
Number of rooms	1	8.84	7.55	10.27	8.22
	2	9.47	7.54	10.14	8.14
	3	9.02	7.52	9.57	8.13
	4 or more	8.13	7.21	8.80	7.47
Presence of outdoor spaces	Yes	8.56	7.36	9.19	7.75
	Partially	8.95	7.26	9.77	8.05
	No	10.22	8.20	10.80	8.69
Daily news hours	Few (less than 1 h)	7.86	6.94	8.60	7.44
	Regularly (2 h)	8.25	6.97	9.02	7.61
	Much (3 or 4 h)	9.51	7.54	10.04	7.67
	All day (4 h or more)	10.95	8.56	11.50	9.22

**TABLE 3:** Results of repeated measures ANOVA for depression.

	**Effect**	**Repeated measures for depression**		
		***F***	***p-*value**	**$\eta_{\text{p}}^{2}$**
All sample	Time	98.84	0.001	0.016
Do you quarantine?	Time	22.60	0.001	0.004
	Group	3.09	0.079	0.001
	Time*Group	0.830	0.362	0.000
Risk factor for COVID-19	Time	20.96	0.001	0.003
	Group	15.53	0.001	0.005
	Time*Group	0.428	0.652	0.000
Age group	Time	52.86	0.001	0.009
	Group	162.97	0.001	0.075
	Time*Group	1.17	0.319	0.001
Gender	Time	52.95	0.001	0.009
	Group	91.99	0.001	0.015
	Time*Group	0.54	0.461	0.000
Educational level	Time	27.33	0.001	0.005
	Group	47.33	0.001	0.030
	Time*Group	2.30	0.057	0.002
Perception of economic impact	Time	43.51	0.001	0.007
	Group	33.36	0.001	0.023
	Time*Group	1.01	0.400	0.001
Work regularly	Time	54.29	0.001	0.009
	Group	59.66	0.001	0.010
	Time*Group	0.842	0.359	0.000
Number of children	Time	26.69	0.001	0.004
	Group	58.27	0.001	0.037
	Time*Group	1.020	0.396	0.001
Alone or accompanied during quarantine	Time	42.31	0.001	0.007
	Group	1.29	0.257	0.000
	Time*Group	1.55	0.213	0.000
Older adults in charge	Time	13.65	0.001	0.003
	Group	26.33	0.001	0.005
	Time*Group	1.21	0.271	0.000
Number of rooms	Time	50.62	0.001	0.008
	Group	12.05	0.001	0.006
	Time*Group	1.387	0.245	0.001
Presence of outdoor spaces	Time	43.16	0.001	0.007
	Group	10.83	0.001	0.004
	Time*Group	0.687	0.503	0.000
Daily news hours	Time	31.69	0.001	0.005
	Group	30.04	0.001	0.019
	Time*Group	1.12	0.344	0.001

Overall, average depression scores increased at the second survey compared to the first one, so it is possible to confirm the existence of a time effect. In most cases, this increase was statistically significant, however, the effect size of these differences were very small or almost imperceptible. It is also possible to confirm the effect of some socio-demographic characteristics, such as having risk factor for COVID-19, age, gender, educational level, perception of economic impact, to have a regular work, the number of children, having older adults in charge, the number of rooms in the quarantine site, the presence of outdoor spaces, and the daily news hours consumed. No interaction effects were observed for depressive symptoms.

### Changes in State Anxiety Between the First and the Second Survey

Descriptive statistics for depressive anxiety levels are presented in Table 4. In the first place, we conducted a repeated measures ANOVA considering all the sample. The statistic showed a significant difference (*F* = 97.61; *p* < 0.001) between T1 and T2, however, the effect size of this difference was very small ($\eta_{\text{p}}^{2}$ = 0.016). In the second place, we conducted the ANOVAs considering the socio-demographic variables as the inter-subject factor and time-point as the intra-subject factor. Results are presented in Table 5.

**TABLE 4:** Descriptive statistics for anxiety in Time 1 and Time 2.

	**Anxiety**	**Time 1**		**Time 2**	
		**ME**	***SD***	**ME**	***SD***
All sample		1.16	0.50	1.11	0.51
Do you quarantine?	Yes	1.15	0.50	1.11	0.51
	Exempted/No	1.22	0.49	1.14	0.51
Risk factor for COVID-19	Yes	1.14	0.50	1.08	0.51
	I don’t know	1.33	0.53	1.24	0.55
	No	1.15	0.50	1.11	0.50
Perceived degree of quarantine compliance	10–40%	1.31	0.53	1.25	0.55
	50–70%	1.25	0.50	1.23	0.51
	80–100%	1.16	0.50	1.11	0.51
Age group	18–25	1.31	0.52	1.27	0.52
	26–40	1.18	0.50	1.13	0.51
	41–60	1.09	0.46	1.05	0.48
	60 or +	0.98	0.50	0.92	0.45
Gender	Female	1.19	0.50	1.13	0.51
	Male	1.04	0.45	1.01	0.47
	Other	1.26	0.59	1.26	0.62
	Prefer not to answer	1.17	0.58	1.09	0.65
Educational level	Postgrad	1.09	0.47	1.05	0.49
	University (complete)	1.15	0.48	1.10	0.50
	University (incomplete)	1.21	0.52	1.16	0.52
	Secondary (complete)	1.26	0.54	1.20	0.56
	Secondary (incomplete)	1.30	0.54	1.23	0.52
	Primary (complete)	–	–	–	–
	Primary (incomplete)	–	–	–	–
Perception of economic impact	No	1.09	0.48	1.05	0.50
	Few	1.16	0.47	1.11	0.48
	Some	1.23	0.48	1.17	0.49
	Much	1.31	0.51	1.25	0.53
	Very much	1.26	0.54	1.21	0.55
Work regularly	Yes	1.15	0.49	1.10	0.50
	No	1.20	0.53	1.15	0.54
Number of children	0	1.19	0.51	1.14	0.52
	1	1.17	0.51	1.12	0.51
	2	1.11	0.47	1.08	0.48
	3	1.09	0.46	1.01	0.47
	4 or more	1.10	0.45	0.99	0.48
Alone or accompanied during quarantine	Alone	1.12	0.50	1.04	0.50
	Accompanied	1.17	0.50	1.12	0.51
Older adults in charge	No	1.14	0.49	1.10	0.50
	Yes	1.30	0.53	1.20	0.54
Number of rooms	1	1.18	0.52	1.10	0.53
	2	1.19	0.50	1.13	0.51
	3	1.17	0.50	1.12	0.50
	4 or more	1.13	0.48	1.10	0.50
Presence of outdoor spaces	No	1.22	0.52	1.15	0.52
	Partially	1.18	0.50	1.13	0.50
	Yes	1.15	0.49	1.10	0.51
Daily news hours	Few (less than 1 h)	1.06	0.47	1.03	0.49
	Regularly (2 h)	1.14	0.46	1.10	0.48
	Much (3 or 4 h)	1.24	0.49	1.17	0.51
	All day (4 h or more)	1.36	0.55	1.27	0.56

**TABLE 5:** Results of repeated measures ANOVA for anxiety.

	**Effect**	**Repeated measures for anxiety**		
		***F***	***p-*value**	**$\eta_{\text{p}}^{2}$**
All sample	Time	97.61	0.001	0.016
Do you quarantine?	Time	52.02	0.001	0.009
	Group	5.96	0.015	0.001
	Time*Group	4.34	0.037	0.001
Risk factor for COVID-19	Time	48.42	0.001	0.008
	Group	12.72	0.001	0.004
	Time*Group	2.84	0.058	0.001
Age group	Time	63.88	0.001	0.010
	Group	70.48	0.001	0.034
	Time*Group	0.93	0.425	0.000
Gender	Time	44.30	0.001	0.007
	Group	83.92	0.001	0.014
	Time*Group	2.87	0.090	0.000
Educational level	Time	27.48	0.001	0.005
	Group	19.35	0.001	0.013
	Time*Group	0.68	0.607	0.000
Perception of economic impact	Time	70.96	0.001	0.012
	Group	35.97	0.001	0.024
	Time*Group	0.721	0.577	0.000
Work regularly	Time	56.89	0.001	0.009
	Group	11.16	0.001	0.002
	Time*Group	0.30	0.584	0.000
Number of children	Time	70.64	0.001	0.012
	Group	11.77	0.001	0.008
	Time*Group	2.20	0.066	0.001
Alone or accompanied during quarantine	Time	84.20	0.001	0.014
	Group	16.29	0.001	0.003
	Time*Group	7.73	0.005	0.001
Older adults in charge	Time	43.84	0.001	0.009
	Group	26.41	0.001	0.005
	Time*Group	4.28	0.039	0.001
Number of rooms	Time	45.36	0.001	0.008
	Group	3.75	0.011	0.002
	Time*Group	1.45	0.227	0.001
Presence of outdoor spaces	Time	48.72	0.001	0.008
	Group	4.19	0.015	0.001
	Time*Group	0.33	0.716	0.000
Daily news hours	Time	112.68	0.001	0.019
	Group	79.60	0.001	0.039
	Time*Group	8.03	0.001	0.004

**FIGURE 1:**
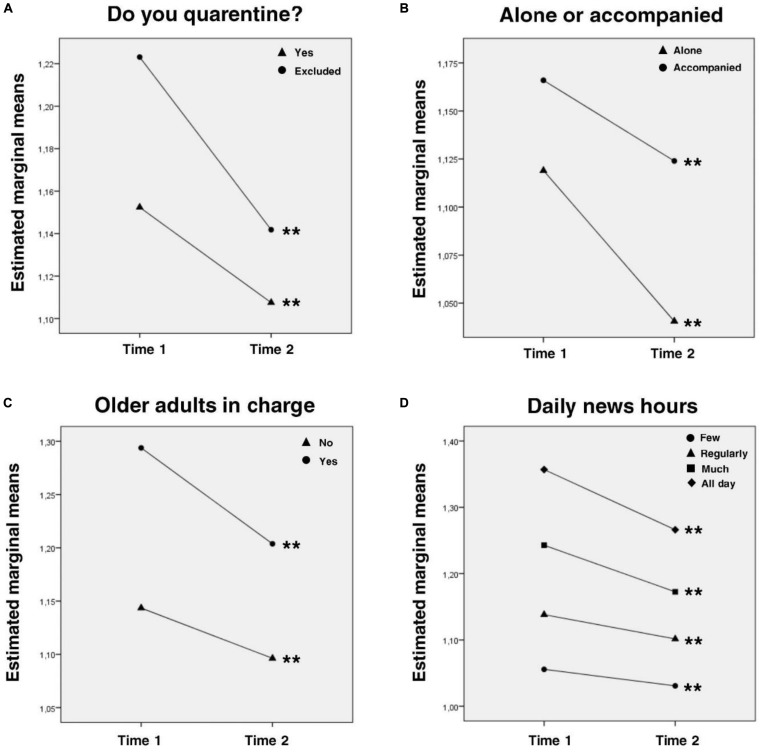
Interaction effects for anxiety levels. *Intragroup* analysis with Bonferroni correction are expressed in the graphs: line-group with ^∗^ showed *p* < 0.05 intragroup differences between time 1 and time 2; line-group with ^∗∗^ showed *p* < 0.01 intragroup differences between time 1 and time 2. *Post hoc intergroup* comparisons with Bonferroni correction (only significant differences are considered, all the comparisons missing were not statistically significant; ^∗^*p* < 0.05, ^∗∗^*p* < 0.01): **(A)** Time 1: Yes, Excluded^∗∗^. **(B)** All the intergroup comparisons were statistically significant^∗∗^. **(C)** All the intergroup comparisons were statistically significant^∗∗^. **(D)** All the intergroup comparisons were statistically significant^∗∗^.

**FIGURE 2:**
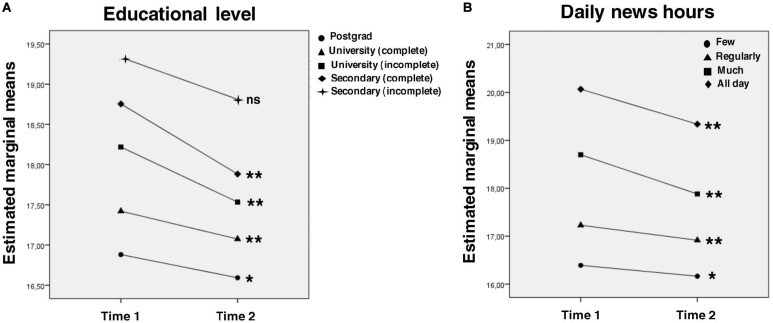
Interaction effects for negative affect. *Intragroup* analysis with Bonferroni correction are expressed in the graphs: line-group with ^∗^ showed *p* < 0.05 intragroup differences between time 1 and time 2; line-group with ^∗∗^ showed *p* < 0.01 intragroup differences between time 1 and time 2; line-group with ^*ns*^ showed no statistical intragroup differences between time 1 and time 2. *Post hoc intergroup* comparisons with Bonferroni correction (only significant differences are considered, all the comparisons missing were not statistically significant; ^∗^*p* < 0.05, ^∗∗^*p* < 0.01): **(A)** Time 1: secondary (incomplete), postgrad^∗^; secondary (complete), university (complete)^∗∗^; secondary (complete), postgrad^∗∗^; university (incomplete), university (complete)^∗∗^; university (incomplete), postgrad^∗∗^. Time 2: secondary (incomplete), postgrad^∗^; secondary (complete), postgrad^∗∗^; university (incomplete), postgrad^∗∗^. **(B)** All the intergroup comparisons were statistically significant^∗∗^.

**FIGURE 3:**
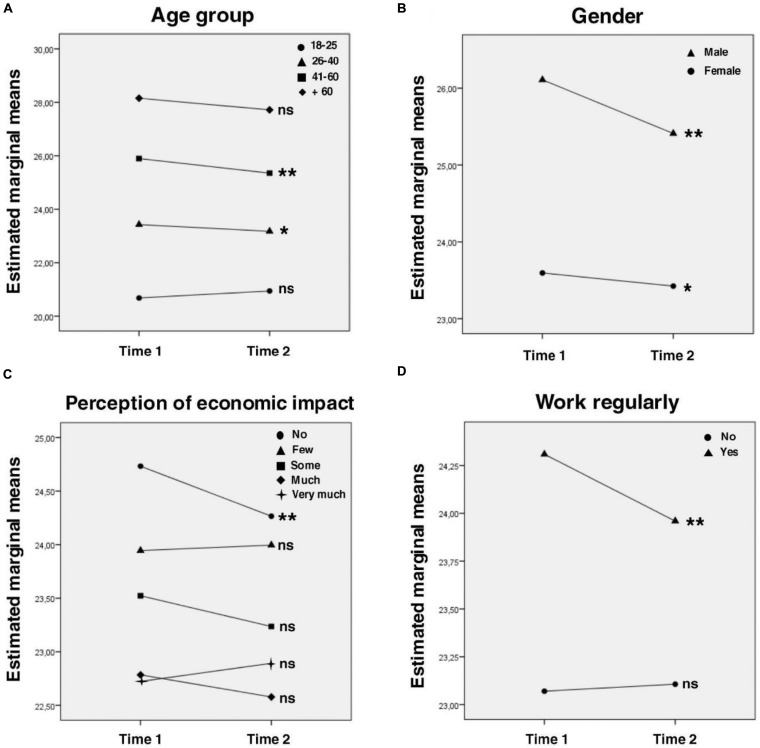
Interaction effects for positive affect. *Intragroup* analysis with Bonferroni correction are expressed in the graphs: line-group with * showed *p* < 0.05 intragroup differences between time 1 and time 2; line-group with ** showed *p* < 0.01 intragroup differences between time 1 and time 2; line-group with ^*ns*^ showed no statistical intragroup differences between time 1 and time 2. *Post hoc intergroup* comparisons with Bonferroni correction (only significant differences are considered, all the comparisons missing were not statistically significant; *p < 0.05, **p < 0.01): **(A)** All the intergroup comparisons were statistically significant**. **(B)** All the intergroup comparisons were statistically significant**. **(C)** Time 1: no, few*; no, some**; no, much**; no, very much**; few, very much**. Time 2: no, some**; no, much**; no, very much**; few, much*; few, very much*. **(D)** All the intergroup comparisons were statistically significant**.

State-anxiety tends to decrease at all the analyzed categories after 2 weeks of quarantine. In most cases, this decrease was statistically significant, but the effect size were very small or almost imperceptible. This would imply that isolation would not increase anxiety but, on the contrary, tends to decrease it. All socio-demographic characteristics showed also effects over anxiety levels. Four interaction effects were also found: (1) essential workers showed higher levels of anxiety at T1 (*p* < 0.001), but larger decrease than non-essential workers. Differences in anxiety between both groups at T2 were non-significant (Figure 1A); (2) people quarantining alone showed less anxiety (both at T1 and T2; *p* < 0.001) and larger decrease compared with people accompanied (Figure 1B); (3) people with elderly dependents obtained higher anxiety scores at both T1 and T2, but they also showed greater decrease compared with people with no older adults in charge (Figure 1C); (4) although those who consumed more news had higher levels of anxiety at both times, the decrease in anxiety levels over time was larger for these groups (and remained more stable for those who consumed less news) (Figure 1D).

### Changes in Negative Affect Between the First and the Second Survey

Descriptive statistics for negative affect are presented in Table 6. In the first place, we conducted a repeated measures ANOVA considering all the sample. The statistic showed a significant difference (*F* = 59.04; *p* < 0.001) between T1 and T2, however, the effect size of this difference was very small ($\eta_{\text{p}}^{2}$ = 0.010). In the second place, we conducted the ANOVAs considering the socio-demographic variables as the inter-subject factor and time-point as the intra-subject factor. Results are presented in Table 7.

**TABLE 6:** Descriptive statistics for negative affect in Time 1 and Time 2.

**Negative affect**		**Time 1**		**Time 2**	
		**ME**	***SD***	**ME**	***SD***
All sample		17.60	6.14	17.14	6.30
Do you quarantine?	Yes	17.56	6.12	17.10	6.29
	Exempted/No	18.16	6.39	17.57	6.48
Risk factor for COVID-19	Yes	17.52	6.16	17.03	6.50
	I don’t know	19.71	6.98	18.55	6.84
	No	17.53	6.08	17.11	6.23
Perceived degree of quarantine compliance	10–40%	18.90	6.93	18.37	5.97
	50–70%	18.05	7.46	18.13	7.48
	80–100%	17.59	6.11	17.11	6.28
Age group	18–25	19.51	6.96	19.05	7.31
	26–40	17.91	6.30	17.35	6.38
	41–60	16.65	5.34	16.36	5.59
	60 or +	15.32	4.73	14.83	4.92
Gender	Female	17.99	6.27	17.50	6.41
	Male	15.92	5.17	15.58	5.47
	Other	19.75	8.98	18.70	9.80
	Prefer not to answer	16.15	5.82	16.50	8.46
Educational level	Postgrad	16.88	5.63	16.59	5.95
	University (complete)	17.42	5.95	17.08	6.03
	University (incomplete)	18.22	6.52	17.53	6.74
	Secondary (complete)	18.75	6.91	17.88	6.99
	Secondary (incomplete)	19.33	7.63	18.81	6.83
	Primary (complete)	–	–	–	–
	Primary (incomplete)	–	–	–	–
Perception of economic impact	No	16.86	5.81	16.46	6.08
	Few	17.65	5.94	17.17	6.16
	Some	18.20	6.07	17.78	6.16
	Much	19.57	6.92	18.71	6.74
	Very much	18.74	6.84	18.17	7.05
Work regularly	Yes	17.46	6.00	17.00	6.16
	No	18.20	6.67	17.72	6.86
Number of children	0	17.98	6.45	17.52	6.70
	1	17.99	6.48	17.56	6.46
	2	17.02	5.49	16.66	5.59
	3	16.48	5.01	15.82	5.14
	4 or more	15.94	4.69	15.36	4.73
Alone or accompanied during quarantine	Alone	16.86	5.86	16.21	6.00
	Accompanied	17.75	6.19	17.32	6.35
Older adults in charge	Yes	19.40	7.39	18.66	7.24
	No	17.42	6.05	16.94	6.27
Number of rooms	1	17.03	5.84	16.69	6.20
	2	17.70	6.21	17.28	6.34
	3	17.86	6.27	17.28	6.38
	4 or more	17.43	6.04	17.00	6.25
Presence of outdoor spaces	Yes	17.56	6.10	17.09	6.28
	Partially	17.69	6.22	17.26	6.38
	No	17.85	6.42	17.47	6.44
Daily news hours	Few (less than 1 h)	16.39	5.62	16.16	5.73
	Regularly (2 h)	17.23	5.63	16.91	6.00
	Much (3 or 4 h)	18.70	6.20	17.88	6.36
	All day (4 h or more)	20.07	7.26	19.34	7.58

**TABLE 7:** Results of repeated measures ANOVA for negative affect.

	**Effect**	**Repeated measures for negative affect**		
		***F***	***p-*value**	**$\eta_{\text{p}}^{2}$**
All sample	Time	59.04	0.001	0.010
Do you quarantine?	Time	22.50	0.001	0.004
	Group	3.94	0.046	0.001
	Time*Group	0.34	0.561	0.000
Risk factor for COVID-19	Time	35.18	0.001	0.006
	Group	10.57	0.001	0.003
	Time*Group	2.69	0.068	0.001
Age group	Time	34.01	0.001	0.006
	Group	73.80	0.001	0.035
	Time*Group	1.28	0.278	0.001
Gender	Time	29.03	0.001	0.005
	Group	112.52	0.001	0.018
	Time*Group	1.01	0.316	0.000
Educational level	Time	17.72	0.001	0.003
	Group	12.74	0.001	0.008
	Time*Group	2.61	0.034	0.002
Perception of economic impact	Time	50.05	0.001	0.009
	Group	27.38	0.001	0.019
	Time*Group	0.82	0.514	0.001
Work regularly	Time	38.62	0.001	0.006
	Group	15.43	0.001	0.003
	Time*Group	0.02	0.880	0.000
Number of children	Time	30.66	0.001	0.005
	Group	17.19	0.001	0.011
	Time*Group	0.384	0.820	0.000
Alone or accompanied during quarantine	Time	43.14	0.001	0.007
	Group	25.01	0.001	0.004
	Time*Group	1.75	0.186	0.000
Older adults in charge	Time	22.23	0.001	0.005
	Group	35.01	0.001	0.007
	Time*Group	1.00	0.317	0.000
Number of rooms	Time	17.32	0.001	0.003
	Group	1.90	0.127	0.001
	Time*Group	0.50	0.686	0.000
Presence of outdoor spaces	Time	21.08	0.001	0.003
	Group	0.83	0.435	0.000
	Time*Group	0.12	0.887	0.000
Daily news hours	Time	64.86	0.001	0.011
	Group	83.56	0.001	0.041
	Time*Group	5.66	0.001	0.003

As it is shown, negative affect decreased in all categories after 2 weeks of isolation. This decrease was statistically significant, but the effect size was very small or almost imperceptible. Almost all socio-demographic factors showed effects over negative affect (except number of rooms in the quarantine site and presence of outdoor spaces). Two interaction effects were observed: (1) at T1, the higher the educational level, the lower the negative affect; at T2, the Postgrad group is the only one that differs significantly from the rest, with lower negative affect; also the group that completed secondary education is the one with larger decrease in negative affect, followed by incomplete university group (Figure 2A); (2) regarding daily news hours, all groups showed a significant decrease in negative affect scores between T1 and T2; and, similar to anxiety levels, the groups who consumed more news showed higher levels of negative affect at T1, but larger decrease over time (Figure 2B).

### Changes in Positive Affect Between the First and the Second Survey

Descriptive statistics for positive affect are presented in Table 8. In the first place, we conducted a repeated measures ANOVA considering all the sample. The statistic showed a significant difference (*F* = 14.47; *p* < 0.001) between T1 and T2, however, the effect size of this difference was close to zero ($\eta_{\text{p}}^{2}$ = 0.002). In the second place, we conducted the ANOVAs considering the socio-demographic variables as the inter-subject factor and time-point as the intra-subject factor. Results are presented in Table 9.

**TABLE 8:** Descriptive statistics for positive affect in Time 1 and Time 2.

**Positive affect**		**Time 1**		**Time 2**	
		**ME**	***SD***	**ME**	***SD***
All sample		24.06	7.67	23.79	7.67
Do you quarantine?	Yes	23.96	7.65	23.70	7.66
	Exempted/No	25.18	7.88	24.75	7.67
Risk factor for COVID-19	Yes	25.29	7.65	25.00	7.76
	I don’t know	22.43	7.60	21.90	7.66
	No	23.83	7.65	23.57	7.61
Perceived degree of quarantine compliance	10–40%	23.80	7.64	23.05	8.43
	50–70%	22.72	7.43	23.03	7.51
	80–100%	24.10	7.67	23.81	7.67
Age group	18–25	20.68	7.10	20.94	7.38
	26–40	23.43	7.46	23.17	7.52
	41–60	25.90	7.51	25.35	7.46
	60 or +	28.15	7.15	27.72	7.15
Gender	Female	23.60	7.50	23.42	7.61
	Male	26.11	8.07	25.41	7.73
	Other	22.35	7.38	21.45	6.97
	Prefer not to answer	24.45	8.33	24.05	6.98
Educational level	Postgrad	25.45	7.91	25.07	7.73
	University (complete)	24.20	7.31	23.89	7.48
	University (incomplete)	22.81	7.59	22.59	7.50
	Secondary (complete)	23.04	8.02	23.16	8.32
	Secondary (incomplete)	24.12	7.50	23.71	8.14
	Primary (complete)	–	–	–	–
	Primary (incomplete)	–	–	–	–
Perception of economic impact	No	24.73	7.67	24.27	7.67
	Few	23.95	7.74	24.00	7.77
	Some	23.52	7.54	23.24	7.45
	Much	22.78	7.05	22.58	7.03
	Very much	22.72	7.68	22.89	7.94
Work regularly	Yes	24.31	7.59	23.96	7.63
	No	23.07	7.94	23.11	7.80
Number of children	0	22.86	7.62	22.78	7.72
	1	24.40	7.45	24.02	7.41
	2	25.46	7.36	24.85	7.31
	3	26.46	7.44	25.98	7.38
	4 or more	27.54	8.11	27.21	7.88
Alone or accompanied during quarantine	Alone	24.52	7.86	24.52	7.96
	Accompanied	23.97	7.63	23.65	7.60
Older adults in charge	Yes	22.92	7.47	22.88	7.47
	No	24.03	7.75	23.77	7.71
Number of rooms	1	23.69	8.07	23.39	8.45
	2	23.08	7.76	22.82	7.67
	3	23.90	7.46	23.69	7.46
	4 or more	24.72	7.63	24.42	7.66
Presence of outdoor spaces	Yes	24.32	7.65	24.04	7.64
	Partially	23.50	7.56	23.23	7.59
	No	22.62	7.99	22.44	8.01
Daily news hours	Few (less than 1 h)	24.76	7.85	24.52	7.87
	Regularly (2 h)	24.09	7.40	23.77	7.47
	Much (3 or 4 h)	23.43	7.54	23.15	7.46
	All day (4 h or more)	22.96	7.64	22.74	7.64

**TABLE 9:** Results of repeated measures ANOVA for positive affect.

	**Effect**	**Repeated measures for negative affect**		
		***F***	***p-*value**	**$\eta_{\text{p}}^{2}$**
All sample	Time	14.47	0.001	0.002
Do you quarantine?	Time	7.06	0.008	0.001
	Group	11.62	0.001	0.002
	Time*Group	0.43	0.510	0.000
Risk factor for COVID-19	Time	6.54	0.011	0.001
	Group	26.20	0.001	0.009
	Time*Group	0.25	0.780	0.000
Age group	Time	6.91	0.009	0.001
	Group	154.82	0.001	0.071
	Time*Group	4.41	0.004	0.002
Gender	Time	22.37	0.001	0.004
	Group	92.33	0.001	0.015
	Time*Group	8.22	0.004	0.001
Educational level	Time	2.37	0.124	0.000
	Group	29.99	0.001	0.019
	Time*Group	0.65	0.624	0.000
Perception of economic impact	Time	2.58	0.108	0.000
	Group	12.71	0.001	0.009
	Time*Group	2.83	0.023	0.002
Work regularly	Time	3.02	0.083	0.000
	Group	20.66	0.001	0.003
	Time*Group	4.60	0.032	0.001
Number of children	Time	12.03	0.001	0.002
	Group	54.39	0.001	0.035
	Time*Group	2.31	0.056	0.002
Alone or accompanied during quarantine	Time	2.89	0.089	0.000
	Group	8.16	0.004	0.001
	Time*Group	2.71	0.100	0.000
Older adults in charge	Time	0.92	0.337	0.000
	Group	6.71	0.010	0.001
	Time*Group	0.51	0.476	0.000
Number of rooms	Time	4.49	0.034	0.001
	Group	16.98	0.001	0.008
	Time*Group	0.07	0.976	0.000
Presence of outdoor spaces	Time	4.89	0.027	0.001
	Group	14.21	0.001	0.005
	Time*Group	0.06	0.940	0.000
Daily news hours	Time	11.75	0.001	0.002
	Group	16.35	0.001	0.008
	Time*Group	0.10	0.961	0.000

All socio-demographic characteristics showed also effects over positive affect, and four interaction effects were found: (1) regarding age, the younger the person, the lower the positive affect, both at T1 and T2; but intragroup differences were only significant for the 26–40 and the 41–60 groups (Figure 3A); (2) positive affect was significantly higher in men, both at T1 and T2; but males showed larger decrease of positive affect than females over time (Figure 3B); (3) regarding economic impact, people who reported no economic impact showed higher positive affect, but larger decrease over time; groups who reported some level of economic impact showed lower positive affect (at both T1 and T2), but remain more stable overt time (Figure 3C); (4) those who work regularly have significantly higher positive affect, both at T1 and T2; but while the group that does not work regularly remained stable over time, the group that works regularly showed a significant decrease in their positive affect (Figure 3D).

## Discussion

In general, after 2 weeks of quarantine, a small size increase in depressive symptoms was observed across the sample. On the contrary, a decrease in anxiety, and negative and positive affect was observed, also with small effect size. So our results show small size differences and some interaction effects (with effect size close to zero). Wang et al. (2020), in the study on the impact of quarantine in China, found almost no difference in the first 2 weeks of lockdown. However, it is important to contextualize the comparison between both studies. Wang et al. (2020) conducted the first survey between January 31 and February 02. At that time, China had about 30,000 confirmed cases of COVID-19. The second survey was conducted 28 days later, between February 28 and March 01, with about 80,000 cases. In contrast, in our study, at the time of the first survey, Argentina had about 500 confirmed cases of COVID-19. Two weeks later, when the second survey was conducted, Argentina has approximately 1,900 cases. This is important since we observed an emotional impact, even though the number of confirmed cases was considerably lower and the time between measures was shorter. The Argentine cultural context and other variables (e.g., the perception of a possible economic crisis in the country), could probably explain these discrepancies.

### Depression

Regarding depression specifically, the symptoms increased very slightly in most groups. We observed a slightly more pronounced increase in depression (although still with small effect size) for those quarantined in a studio apartment (1 room), compared to people who had more rooms in the house. This suggests that the physical features of the quarantine location may affect people’s mood. There was also a slightly larger increase in depressive symptoms in those with postgraduate education and in those who have regular works (compared to those who are unemployed). This may suggest people who are usually more proactive or engaged in different activities are most affected by being isolated or inactive.

### Anxiety

Anxiety levels showed a slight decrease in the full sample. Specifically, those exempted from quarantine (workers of essential services) showed a larger decrease than those quarantining. This may be due to several factors. Firstly, essential workers continued with their routines, so this group probably continue living in a sort of “normality” context. On the other hand, and since we don’t have a pre-lockdown measure, it is possible that anxiety levels had increased greatly at T1 in essential workers in the face of the uncertainty of the situation, but decreased more rapidly in the absence of significant changes in daily life. It is also important to highlight that there is a lot of variability in this group: as mentioned before, around 44 essential activities could be counted (health workers, security forces, personnel business employees, people from the agricultural sector among others). This variability makes it very difficult to find a single explanation for the larger decrease in anxiety levels in essential workers.

Lower anxiety levels were found among people quarantining alone compared to people accompanied. A possible explanation is that those who quarantine alone avoid some relationship and cohabitation problems that can be exacerbated in the context of confinement. It would be necessary to further explore this group since it is not the same to be alone during isolation than to be a person with a certain trait of social isolation in general.

Regarding higher anxiety levels in people with elderly dependents, this probably occurs because this is an at-risk population. Also, the larger decrease in this group may be due to the fact that after 14 days the confirmed cases and deaths in the country did not increase noticeably. Therefore, people may have felt more in control of the situation by reducing exposure to the virus.

Finally, regarding daily news hours, we found higher anxiety in people with more news consumption. The evidence suggests that sustained exposure to the media can lead to increased anxiety and stress (Brooks et al., 2020). Also, the larger decrease in anxiety in the group that consumes a lot of news may be due to the fact that constant exposure produces habituation and, consequently, the same stimulus does not produce the same response as at the beginning. Also, a pre-lockdown measured would have been clarifying in this matter.

### Negative Affect

Concerning negative affect, it decreased very slightly. Since the first assessment was made when the isolation measures had already started (and we do not have a pre-quarantine assessment), it is possible that negative emotions grew higher during the first days of quarantine, but slowly decrease as people get used to the new situation. Regarding educational level, the group with complete secondary education is the one that showed the larger decrease of their negative affect between T1 and T2 (followed by incomplete university). In T2, the postgrad group is the only one that differs significantly from the rest, with lower negative affect. It seems that having a higher educational level could be a protective factor. These results are consistent with those presented by Bracke et al. (2014), Brooks et al. (2020), and Moreira et al. (2020) and differ from those found in other population contexts, where higher educational levels were associated with more symptoms (Qiu et al., 2020). This could be due to the fact that people with a higher level of education may have a more informed and adjusted view of the situation and, therefore, entail lower levels of concern.

### Positive Affect

About positive affect, it tends to decrease very slightly. In relation to age, the younger the person is, the lower the positive affect. It is common for young people to present and experience less positive affect than older people. Different studies have shown a tendency for older people to regulate emotions more effectively than younger people, keeping positive feelings active and avoiding negative ones (American Psychological Association [APA], 2005). On the other hand, many of the young people in the sample reported incomplete university studies, so it is possible that there are many students among them and that the initial suspension of academic activities resulted in feelings of relief and calm.

About gender differences, although males reported higher positive affect, they showed a larger decrease than females over time. This may be due to the change in their routines, the increase in the number of hours at home, and sharing roles of parenting and caring for those who might not be used to it (Cerrato and Cifre, 2018).

Regarding economic impact, the trend is: the lower the economic impact, the more the positive affect at both times. Also, people who reported no economic impact showed higher positive affect, but larger decrease over time. The largest decrease may be due to the fact that the people who had no economic impact are also the people who continued working. Adjusting to teleworking (for teachers for example) and matching its demands with the household’s daily demands can be the cause of these results. This is consistent with the interaction effect found among people who reported working regularly as well: higher positive affect, but larger decrease. On the other hand, question about economic impact were asked at the beginning of isolation (T1), so the perception in relation to the economic impact may have changed.

The slight increase in the levels of depressive symptoms is consistent with the decrease in positive affect (e.g., enthusiasm, interest). However, the levels of negative affect also showed a slight decrease. This could indicate that the increase in BDI means is not caused by changes in mood but rather by changes in the daily habits that the instrument explores (e.g., diet and sleep). In fact, some studies have already reported that there are changes in daily habits as a consequence of isolation measures. For example, some studies indicate that the COVID-19 pandemic appear to be a risk factor for sleep disorders (Barrea et al., 2020; Casagrande et al., 2020). Other studies have also reported changes in diet and weight gain during quarantine (Di Renzo et al., 2020). However, the differences showed very small effect size. Further evaluation over time may alter this result.

Accordingly with various international organizations, we understand health as an integrative construct, so we emphasize the importance of considering the psychological effects of quarantine when making decisions. We hope that the preliminary information provided in this study will contribute to generating clear and useful public policies, in the short, medium and long term. These actions should aim at minimizing the negative effect of mandatory isolation on mental health. There is no doubt that quarantine and social isolation has been one of the best preventive measures, and has been widely recommended by experts to stop the spread of the virus. However, while quarantine has proven to be effective, as time goes by, it seems that the consequences for mental health are getting worse: loneliness, reduced social and physical contact, confinement, lack of privacy, loss of daily routines, etc. can also lead to illness and carry significant costs at the psychological, physical, and social levels. The sustained stress response over time, such as that which can be expected in this situation, has a negative impact not only on mental health but also on the immune system (Grant et al., 2009), making people more vulnerable both to the transmission of COVID-19 and to other illness that require medical care and the use of health resources.

The data presented in this study provide empirical evidence that mandatory quarantine has a psychological effect on the population, especially on certain groups. Although the effect sizes were small, and although it is not possible to anticipate what will happen with the pandemic in the future previous research (Brooks et al., 2020) suggests that symptoms of post-traumatic stress may arise in people who have been quarantined in the long term. Hence, sustaining these measures in the long term could lead to a greater effect on mental health. Without effective prevention actions, this could become a public health problem and negatively impact productivity.

Our study has two great strengths. The first is to have worked on a large sample of general population. The second is to have carried out a longitudinal follow-up of the emotional impact of the quarantine. At present, there is only a few studies that have conducted similar follow-up (e.g., Wang et al., 2020, in China, with a considerably smaller sample). Although this study has some limitations, one of the main ones is that most of the sample was composed of people with university studies. This represents a limit to the generalization of the results, and further studies should try to reach those people with lower educational levels. In addition, the study has no pre-quarantine assessment, which would have been extremely enriching. Finally, since quarantine measures are still in place, further assessments (including a post-quarantine assessment) are needed to assess long-term effects of isolation on mental health.

Given the findings reported here, it seems reasonable to make the following recommendations. It is necessary to keep monitoring of anxiety levels, depressive symptoms, emotional distress and other mental health-related aspects in the general population. It is also necessary to provide official information about the spread of the COVID-19, and specifically about the issues that appear to be of most concern to the population (e.g., the impact of the disease on public health and on the national economy). It is important to discourage excessive consumption of news, and the reproduction of false and/or biased information. Finally, it is also essential to create programs aimed at promoting mental health in the population, and to distribute information on this subject, encouraging habits associated with greater well-being (such as maintaining a healthy diet, healthy routines, daily physical and intellectual activity, etc.) and discouraging maladaptive behaviors (such as substance abuse, poor nutrition, excessive use of technology, or excessive news consumptions).

## Data Availability Statement

The raw data supporting the conclusions of this article will be made available by the authors, without undue reservation, to any qualified researcher.

## Ethics Statement

The studies involving human participants were reviewed and approved by Bioethics Committee – National University of Mar del Plata. The patients/participants provided their written informed consent to participate in this study.

## Author Contributions

All authors listed have made a substantial, direct and intellectual contribution to the work, and approved it for publication.

## Conflict of Interest

The authors declare that the research was conducted in the absence of any commercial or financial relationships that could be construed as a potential conflict of interest.
